# Mediterranean diet and health in the elderly

**DOI:** 10.3934/publichealth.2023040

**Published:** 2023-07-11

**Authors:** Domenico Giuffrè, Angelo Maria Giuffrè

**Affiliations:** 1 Biologist nutritionist - Via Vespia, 51. Reggio Calabria, 89135, Italy; 2 Department AGRARIA, University of Studies ‘*Mediterranea*’ of Reggio Calabria, 89124, Italy

**Keywords:** Mediterranean diet, elderly, lifestyle, scientific evidence, cardiovascular disease, cancer, diabetes

## Abstract

The Mediterranean diet has probably been the most studied diet since the early 1950s. American physiologist Ancel Keys coined the term since it was based on the dietary habits of those populations bordering the Mediterranean basin, particularly the island of Crete and southern Italy. The motivation for the early studies lay in understanding why these populations had greater longevity and lower occurrence of chronic-degenerative diseases and forms of cancer when compared with the peoples of Northern Europe and North America. Traditionally, this dietary regimen was based on the seasonality of foods and the consumption of unrefined grains, legumes, fish, vegetables, fruits, little meat and use of olive oil as a condiment. The purpose of this paper is to understand, based on current scientific knowledge, how the different nutrients present in such a diet can play a preventive role in the onset of today's most frequent diseases.

## 1. Introduction

The Mediterranean diet (MD) historically refers to a dietary lifestyle, which began to be studied in the early 1950s, typical of many countries bordering the Mediterranean Sea, mainly Southern Italy, Spain and Greece. Willett et al. in their work [1] presented scientific evidence, still valid today, that makes this dietary regimen ideal both for preventive purposes and to support appropriate pharmacological intervention.

The key components on which such a diet has historically been based are, in brief, the following [2]:

- fruits and vegetables in large quantities;

- whole grains;

- legumes and nuts;

- olive oil;

- yogurt and cheese (the latter in moderate amounts);

- eggs (maximum of 4 a week);

- red meat (a few times a week and in small amounts);

- fish and white meat (small amounts);

- wine in low amounts.

Such a diet appears to be normolipidic (25%–35% of total calories), but one feature that differentiates it from the typical diets of Northern European or North American countries is the limited introduction of saturated fatty acids [3] and a ratio of monounsaturated to saturated in favor of the first [4].

The substitution of meat as a protein food by fish obviously results in an increased intake of omega-3 fatty acids whose preventive action against cardiovascular disease is universally recognized [5]. The use, at least three times a week, of legumes is certainly integral to the Mediterranean diet; these foods, in addition to providing a good amount of soluble and insoluble dietary fiber and complex carbohydrates, are rich in proteins of medium biological value without the presence of saturated fats. Traditionally, the methionine deficiency of legumes is compensated for by combining them with cereals which allows the complete amino acid intake to be achieved.

The benefit of such a dietary regimen resides in its ability to maintain health and improve longevity, as declared in 2010 by the United Nations Educational, Scientific and Cultural Organization (UNESCO) [6]. Several benchmark indices such as the Mediterranean diet scale [7] and the Mediterranean adequacy index [8] have been developed in order to validate the results obtained from the numerous studies that relate the Mediterranean diet to the prevention or treatment of various diseases such as metabolic syndrome, diabetes, cardiovascular diseases, neurodegenerative diseases and cancer.

The present work is a narrative review of studies related to the benefits of the Mediterranean diet and the main diseases in the elderly. The bibliography was extracted from SCOPUS database and other databanks.

## 2. Mediterranean diet: Prevention and treatment of major chronic degenerative diseases

### 2.1. Mediterranean diet and Diabetes

Eating habits that have become ever more widespread in recent decades with increased consumption of energy-dense foods that are low in beneficial nutrients and increasingly sedentary lifestyles are some of the established causes that have resulted in Europe reaching rates of overweight and obesity that are lower only than those recorded in North America World Health Organization (WHO) European Regional Office - Obesity Report 2022 [9].

The document shows that 59% of European adults and nearly one out of every three children (29% of boys and 27% of girls) are overweight or affected by obesity. Overweight and obesity are among the leading causes of death and disability in the WHO European region and recent estimates suggest that they cause more than 1.2 million deaths per year, corresponding to more than 13% of total mortality in the Region.

Obesity increases the risk of cardiovascular disease, type 2 diabetes mellitus (T2DM), chronic respiratory disease and cancers such as those of the uterus, breast, colon, prostate and kidney [10]. Abdominal obesity accompanied by dyslipidemia, hypertension and insulin resistance result in metabolic syndrome (MetS) that has been increasing significantly in recent decades. The relationship between obesity and T2DM is so strong that the new term “diabesity” has been introduced [11].

Following the Mediterranean diet is greatly beneficial in the prevention of type 2 diabetes mellitus and allows diabetics to better control their blood glucose. Type 2 diabetic patients who adhere closely to the Mediterranean diet have lower levels of glycosylated hemoglobin (Hba1c) and postprandial blood glucose [12]–[13]. The American Diabetes Association and American Heart Association recommend the Mediterranean diet to improve glycemic control and cardiovascular risk factors in type 2 diabetes. The mechanisms appear to be related to an antioxidant and anti-inflammatory action peculiar to the phytonutrients which the cornerstone foods of the Mediterranean diet are rich in. Glycemic control is also related to the introduction of fiber with the regular use of whole grains, vegetables, fruits and legumes [14].

It was found that when compared with non-diabetic subjects, patients with T2D showed a worse response to cognitive tests, visual memory, verbal memory and motor function. In addition, pre-diabetes was also found to be positively correlated with dementia in the elderly [15].

Studies have demonstrated the relationship between the Mediterranean diet and a better gait speed in non-diabetic older adults and a general attenuation in mobility decline [16].

### 2.2. Mediterranean diet and cardiovascular diseases (CVD)

Diet plays a central role in the occurrence of cardiovascular diseases. A high quantity of sugars, sodium and saturated fatty acids can promote hypertension and obesity which negatively influence the cardiovascular apparatus [17]–[19].

One of the most interesting projects to evaluate the beneficial effects of the Mediterranean diet was PREDIMED which was conducted in Spain between 2003 and 2011and involved adults with high cardiovascular risk. The three groups of study subjects followed, respectively, a Mediterranean diet supplemented with extra virgin olive oil (4 tbsp/day (50 gr/day)); a Mediterranean diet with nuts (30 g/day); and a low-fat diet. The study showed how the first two groups presented a 30 percent decrease in the risk of cardiovascular events [20] and benefits such as improved lipid status, decreased inflammatory status and reduced insulin resistance [21] in comparison with the control group.

Normally at the mitochondrial level, our cells produce reactive oxygen forms (ROS) such as superoxide anion (O^2−^), hydrogen peroxide and hydroxyl radical (•OH) [22]. Such oxygen free radicals, if not properly counterbalanced by antioxidant action by the body, generate harmful oxidative stress to the cells and tissues of our body triggering reactions with other biomolecules such as DNA and lipids. This is one of the causes of chronic degenerative diseases including atherosclerosis and cancer. The defense mechanisms of our cells are carried out by enzymes such as catalase, superoxide dismutase (SOD) and glutathione peroxidase (GPS) for whose cellular production the intake of minerals (iron, copper, selenium and zinc) is indispensable. Additional help in carrying out antioxidant action is played by vitamins A, C and E, phytonutrients such as flavonoids, lutein and lycopene which are found in foods such as watermelon, apples, grapes, red fruits, tomatoes, and green leafy vegetables which in addition are also sources of minerals. Therefore, the dietary intake of such biomolecules is extremely important for preventing and treating cardiovascular disorders [23]. Concerning omega-3 fatty acids, a daily intake of 500 mg of eicosatetraenoic and docosahexaenoic acid and 10 g of linoleic acid is recommended [24]. Linoleic acid is contained in vegetable oils such as olive oil (13%–18%) [25], peanut oil (29%–35%) [26], sesame oil (44%–47%) [27], sunflower oil (32%–35%) [28] and tomato seed oil (47%–61%) [29]. The linoleic content is influenced by many factors such as cultivar and thermal treatments [24]–[29]. The inclusion of fish in the diet (1–3 times a month) was found to reduce the risk of an ischemic stroke by 15% and consumption of 1–4 times a week was found to reduce this risk by 32% [30].

### 2.3. Mediterranean diet and forms of cancer

After cardiovascular disease, as highlighted by GLOBOCAM estimates [31], cancer is the second cause of death and disability in much of the world (Asian countries, Europe, North America). This 2020 data shows that after breast cancer, the most prevalent forms of cancer are lung, colorectal and prostate cancer. According to the PURE study in high- and middle-income countries the incidence of these diseases has already surpassed that of cardiovascular disease [32]. Numerous studies in recent years have highlighted the correlation between a proper Mediterranean diet and the prevention of these forms of cancer [33].

The introduction of polyphenols into the diet seems able to reduce breast cancer and its recurrence. Many of the biological actions of polyphenols have been attributed not only to their antioxidant properties but also to their pleiotropic effect by being able to influence different metabolic pathways [34]. The action carried out by polyphenols can delay and reduce carcinogenic processes in breast tissue [35]–[36]. Among polyphenols, the action of oleuropein, found in both olive fruits and leaves, is of interest for its antiproliferative action on breast cancer cells [37].

With reference to colorectal cancer, we report the study carried out in Spain on around 41000 subjects aged between 29 and 69 years which aimed to evaluate the preventive effects of diet against the onset of this disease. The study was based on a comparison of three different dietary patterns identified as: Western (with high intakes of both saturated fats from dairy products and red meat, refined grains, caloric drinks and sweets), Prudent (with use of light cheeses, vegetables, fruits, whole grains and juices) and Mediterranean (with consumption of fish, legumes, vegetables, whole grains, boiled potatoes, fruit, olive oil and low consumption of juices). The study [38] found both a protective action of the Mediterranean dietary pattern and an increased risk with the Western dietary pattern while the prudent pattern did not seem to influence the frequency of occurrence of this cancer [39]. The protective action of the Mediterranean diet may be related to several factors: the increased introduction of antioxidant nutrients that have a DNA-protective action by counteracting the oxidative power of free radicals [40]; intestinal regularity as a consequence of an increased introduction of dietary fiber [41]; and the creation of a favorable microbiota [42]–[43].

With regard to prostate cancer, although following the Mediterranean diet may reduce its incidence, the curative aspect has not been well delineated [44]. It is reported that the antioxidant and hypolipidemic action may be of benefit to individuals with localized prostate cancer [45]. However, it is well established that a Western diet rich in processed foods and high in calories, may be one of the causes of the increase in this disease.

### 2.4. Mediterranean diet and Alzheimer's and cognitive decline

The rise in the elderly population is increasingly evident not only in industrialized countries but also in developing countries. The increase in life expectancy is leading in parallel not only to an increased presence of chronic-degenerative diseases but also neurological problems such as Alzheimer's disease or cognitive impairment.

Can the Mediterranean diet play a preventive role against such diseases? The predisposing factors for Alzheimer's disease, currently the most common cause of dementia, include the following:

- the deficiency of antioxidant agents such as vitamins. C, E, B_6_, B_12_ and folate [46]–[48];

- the excessive introduction of saturated fatty acids at the expense of mono- and polyunsaturated [49];

- the excessive introduction of simple sugars resulting in increased glycaemia, such that Type II Diabetes mellitus becomes a predisposing factor [50]–[51];

- energy and nutritional malnutrition, often linked to the onset of the disease itself [52] which can damage the appetite control center.

Although studies conducted on the Mediterranean diet and its possible link to a decreased incidence of Alzheimer's disease are not numerous, it may be considered that based on food variety and the introduction of the above-mentioned nutrients such a diet could play a preventive role.

Also, for cognitive impairment the study carried out on 1864 elderly people living in Greece whose adherence to the Mediterranean diet was assessed by using the Mediterranean dietary score (MedDietScore) [53] showed that adherence to this diet may have a beneficial effect [54].

Felisatti et al. associated the Mediterranean diet with higher brain glucose metabolism in the medial temporal lobe and higher performance on attention tests in APOE4 which is the main genetic risk factor for Alzheimer's disease [55].

Gaynor et al., showed that the Mediterranean Diet may support cognitive function despite changes in brain connectivity [56].

Shannon et al. accessed on the UK Biobank, an updated study of more than half a million participants, reported the results for an investigation into disease in middle and older age. They found that the higher the adherence to a MedDiet the lower the risk of dementia independent of genetic factors [57].

## 3. Conclusions

Extensive research over the past seventy years has shown that the preventive action of the Mediterranean diet together with pharmacological treatment protects against most of the chronic-degenerative diseases found in old age. However, in recent decades the principles on which that diet was based have been lost even in Mediterranean countries in favor of the consumption of ultra-processed, high-calorie and low-fiber foods.

The choices made by the governments both of high-income and of developing countries should encourage the production and consumption of those foods that characterized the original Mediterranean diet, a choice which would be reflected in a decrease in health care spending.

## References

[1] Willett WC, Sacks F, Trichopoulou A (1995). Mediterranean diet pyramid: a cultural model for healthy eating. Am J Clin Nutr.

[2] Poli A, Marangoni F, Paoletti R (2008). Non-pharmacological control of plasma cholesterol levels. Nutr Metab Cardiovasc Dis.

[3] Kromhout D, Keys A, Aravanis C (1989). Food consumption patterns in the 1960s in seven countries. Am J Clin Nutr.

[4] Willett C (2006). The Mediterranean diet: science and practice. Public Health Nutr.

[5] Mori TA (2017). Marine OMEGA-3 fatty acids in the prevention of cardiovascular disease. Fitoterapia.

[6] Medina FX (2009). Mediterranean diet, culture and heritage: challenges for a new conception. Public Health Nutr.

[7] Benetou V, Trichopoulou A, Orfanos P (2008). Conformity to traditional Mediterranean diet and cancer incidence: the Greek EPIC cohort. Br J Cancer.

[8] Alberti-Fidanza A, Fidanza F (2004). Mediterranean Adequacy Index of Italian diets. Public Health Nutr.

[9] World Health Organization (WHO) (2022). European Regional Office - Obesity Report 2022.

[10] De Pergola G, Silvestris F (2013). Obesity as a major risk factor for cancer. J Obes.

[11] García Vega D, Ramón González J, Eiras S (2022). Diabesity in elderly cardiovascular disease patients: Mechanisms and regulators. Int J Mol Sci.

[12] Martínez-González MA, de la Fuente-Arrillaga C, Nunez-Córdoba JM (2008). Adherence to Mediterranean diet and risk of developing diabetes: prospective cohort study. BMJ.

[13] Esposito K, Maiorino MI, Bellastella G (2017). Mediterranean diet for type 2 diabetes: cardiometabolic benefits. Endocrine.

[14] Venn BJ, Mann JI (2004). Cereal grains, legumes and diabetes. Eur J Clin Nutr.

[15] Palta P, Schneider AL, Biessels GJ (2014). Magnitude of cognitive dysfunction in adults with type 2 diabetes: a meta-analysis of six cognitive domains and the most frequently reported neuropsychological tests within domains. J Int Neuropsychol Soc.

[16] McClure R, Villani A (2019). Greater adherence to a Mediterranean Diet is associated with better gait speed in older adults with type 2 diabetes mellitus. Clin Nutr ESPEN.

[17] Wang L, Manson JE, Forman JP (2010). Dietary Fatty Acids and the Risk of Hypertension in Middle-Aged and Older Women. Hypertension.

[18] Pikilidou M, Scuteri A, Morrell C (2013). The burden of obesity on blood pressure is reduced in older persons: The SardiNIA study. Obesity.

[19] Appel LJ, Espeland MA, Easter L (2001). Effects of reduced sodium intake on hypertension control in older individuals: Results from the Trial of Nonpharmacologic Interventions in the Elderly (TONE). Arch Intern Med.

[20] Estruch R, Ros E, Salas Salvado J (2013). Primary prevention of cardiovascular disease with a Mediterranean diet. N Engl J Med.

[21] Salas Salvado J, Bullo M, Babio N (2011). Reduction in the incidence of type 2 diabetes with the Mediterranean diet: results of the PREDIMED-Reus nutrition intervention randomized trial. Diabetes Care.

[22] Mailloux RJ (2020). An Update on Mitochondrial Reactive Oxygen Species Production - Antioxidants.

[23] Hertog MG, Kromhout D, Aravanis C (1995). Assunzione di flavonoidi e rischio a lungo termine di malattia coronarica e cancro nello studio dei sette paesi. Arch Intern Med.

[24] Djuricic I, Calder PC (2021). Beneficial Outcomes of Omega-6 and Omega-3 Polyunsaturated Fatty Acids on Human Health: An Update for 2021. Nutrients.

[25] Giuffrè AM, Zappia C, Capocasale M (2017). Effects of high temperatures and duration of heating on olive oil properties for food use and biodiesel production. J Am Oil Chem Soc.

[26] Giuffrè AM, Tellah S, Capocasale M (2016). Seed oil from ten Algerian peanut landraces for edible use and biodiesel production. J Oleo Sci.

[27] Anastasi U, Sortino O, Tuttobene R (2017). Agronomic performance and grain quality of sesame (*Sesamum indicum* L.) landraces and improved varieties grown in a Mediterranean environment. Genet Resour Crop Evol.

[28] Giuffrè AM, Capocasale M, Zappia C (2017). Influence of high temperature and duration of heating on the sunflower seed oil properties for food use and bio-diesel production. J Oleo Sci.

[29] Giuffrè AM, Capocasale M (2016). Physicochemical composition of tomato seed oil for an edible use: the effect of cultivar. Int. Food Res. J.

[30] Mozaffarian D, Longstreth WT, Rozenn N (2005). Fish Consumption and Stroke Risk in Elderly Individuals. The Cardiovascular Health Study. Arch Intern Med.

[31] Sung H, Ferlay J, Siegel RL (2021). Global Cancer Statistics 2020: GLOBOCAN Estimates of Incidence and Mortality Worldwide for 36 Cancers in 185 Countries. CA Cancer J Clin.

[32] Dagenais GR, Leong DP, Rangarajan S (2020). Variations in common diseases, hospital admissions, and deaths in middle-aged adults in 21 countries from five continents (PURE): a prospective cohort study. Lancet.

[33] Donaldson MS (2004). Nutrition and cancer: A review of the evidence for an anti-cancer diet. Nutr J.

[34] Maraldi T, Vauzour D, Angeloni C (2014). Dietary polyphenols and their effects on cell biochemistry and pathophysiology 2013. Oxid Med Cell Longev.

[35] Varinska L, Gal P, Mojzisova G (2015). Soy and breast cancer: Focus on angiogenesis. Int J Mol Sci.

[36] Yang CS, Lambert JD, Sang S (2009). Antioxidative and anti-carcinogenic activities of tea polyphenols. Arch Toxicol.

[37] Messeha SS, Zarmouh NO, Asiri A (2020). Gene expression alterations associated with oleuropein-induced antiproliferative effects and s-phase cell cycle arrest in triple-negative breast cancer cells. Nutrients.

[38] Castelló A, Rodríguez Barranco M, Fernández de Larrea N (2022). Adherence to the western, prudent and mediterranean dietary patterns and colorectal cancer risk: Findings from the Spanish cohort of the European prospective investigation into cancer and nutrition (EPIC-Spain). Nutrients.

[39] Castelló A, Amiano P, de Larrea NF (2018). Low adherence to the western and high adherence to the Mediterranean dietary patterns could prevent colorectal cancer. Eur J Nutr.

[40] Fang YZ, Yang S, Wu G (2022). Free radicals, antioxidants, and nutrition. Nutrition.

[41] Giovannucci E, Willett WC (1994). Dietary factors and risk of colon cancer. Ann Med.

[42] Turnbaugh PJ, Ridaura VK, Faith JJ (2009). The Effect of Diet on the Human Gut Microbiome: A Metagenomic Analysis in Humanized Gnotobiotic Mice. Sci Transl Med.

[43] Moszak M, Szulińska M, Bogdański P (2020). You Are What You Eat—The Relationship between Diet, Microbiota, and Metabolic Disorders—A Review. Nutrients.

[44] Lozano-Lorca M, Rodríguez González M, Salcedo-Bellido I (2022). Dietary Patterns and Prostate Cancer: CAPLIFE Study. Cancers.

[45] Cass S, Wargo J, Pettaway C (2022). A novel Mediterranean dietary intervention for prostate cancer. Cancer Res.

[46] Mielech A, Puścion-Jakubik A, Markiewicz Żukowska R (2020). Vitamins in Alzheimer's disease - Review of the latest reports. Nutrients.

[47] Mecocci P, Polidori MC (2012). Antioxidant Clinical Trials in Mild Cognitive Impairment and Alzheimer's Disease. Biochim Biophys Acta.

[48] Agnew Blais JC, Wassertheil Smoller S, Kang JH (2015). Folate, Vitamin B-6, and Vitamin B-12 intake and mild cognitive impairment and probable dementia in the women's health initiative memory study. J Acad Nutr Diet.

[49] Laitinen MH, Ngandu T, Rovio S (2006). Fat Intake at Midlife and Risk of Dementia and Alzheimer's Disease: A Population-Based Study. Dement Geriatr Cogn Disord.

[50] Morris JK, Vidoni ED, Hone R.A (2014). Impaired Glycemia Increases Disease Progression in Mild Cognitive Impairment. Neurobiol Aging.

[51] Huang CC, Chung CM, Leu HB (2014). Diabetes mellitus and the risk of Alzheimer's disease: A nationwide population-based study. PLoS ONE.

[52] Sergi G, De Rui M, Coin A (2013). Weight Loss and Alzheimer's Disease: Temporal and Aetiologic Connections. Proc Nutr Soc.

[53] Panagiotakos DB, Pitsavos C, Stefanadis C (2006). Dietary patterns: a Mediterranean diet score and its relation to clinical and biological markers of cardiovascular disease risk. Nutr Metab Cardiovasc Dis.

[54] Anastasiou CA, Yannakoulia M, Kosmidis MH (2017). Mediterranean diet and cognitive health: Initial results from the Hellenic longitudinal investigation of ageing and diet. PloS one.

[55] Felisatti F, Chauveau L, de Flores R (2022). Interaction between APOE4 and lifestyle on neuroimaging biomarkers and cognition in cognitively unimpaired older adults. Alzheimer's Dement.

[56] Gaynor AM, Varangis E, Song S (2022). Diet moderates the effect of resting state functional connectivity on cognitive function. Sci Rep.

[57] Shannon OM, Ranson JM, Gregory S (2023). Mediterranean diet adherence is associated with lower dementia risk, independent of genetic predisposition: Findings from the UK Biobank prospective cohort study. BMC Med.

